# The influence of occupational values on college students’ willingness to apply for civil servants: The mediating role of political efficacy

**DOI:** 10.3389/fpsyg.2022.1020863

**Published:** 2022-10-17

**Authors:** Jianchao Ni, Yuanyi Shen, Chunmei Chen, Xiangfu Liu

**Affiliations:** ^1^School of Aerospace Engineering, Xiamen University, Xiamen, China; ^2^Teachers College, Jimei University, Xiamen, China

**Keywords:** occupational values, willingness, civil servants, political efficacy, public service motivation

## Abstract

At present, civil servant positions have become the main employment target of many college students in China, and there is a deep motivation behind this phenomenon. This research conducted an empirical study on college students in 2022 with 566 valid data by using the convenience sampling method. The occupational values scale, political efficacy scale, and the willingness to apply for civil servants scale were used. Descriptive statistical analysis, correlation analysis, and common method deviation were performed by SPSS 26.0 to test the reliability of each scale and the discriminant validity of variables. In addition, the structural equation model and bias-corrected bootstrap were used to explore the influence of occupational values on college students’ willingness to apply for civil servants and analyze the mediating role of political efficacy. The results show that: (1) The dimensions of career stability, prestige value, and public service motivation of occupational values have significant positive impacts on college students’ willingness to apply for civil servants, among which public service motivation has the most significant impact. (2) Occupational values have significant positive impacts on college students’ political efficacy. (3) Political efficacy has a significant positive impact on the college students’ willingness to apply for civil servants. (4) Political efficacy plays a partial mediating role in the transmission path of occupational values and willingness to apply for civil servants. These findings further clarify the logical relationship between occupational values and civil servants’ willingness, providing a theoretical basis and practical reference for college educators to implement college students’ career education.

## Introduction

In recent years, the phenomenon of “civil servant fever” in China has attracted much attention from all sectors of society. Since the first National Examination in China was organized by the former Ministry of Personnel in 1994, the number of applicants for the National Examination has risen exponentially. Initially, there were only more than 4,400 applicants. In 2009, the number of applicants exceeded one million for the first time. In October 2021, a total of 2.123 million people passed the qualification examination for the 2022 National Civil Service Examination, 2021. The number of applicants for the national examination exceeded the 2 million mark for the first time. The driving force behind this phenomenon has become the focus of many scholars.

Deci and Ryan (2000) hold that people’s behavior is goal-oriented. Moreover, people’s goals are different based on the self-determination theory in psychology. Among various goals, it is recognized that the reason of choice might come from desirable outcomes, such as higher income. The traditional normative theory assumes that people are rational decision-makers and they are mainly driven by self-interest (Neumann and Morgenstern, 1944; Luce and Raiffa, 1957). O’Riordan (2013) points out that public service can attract employees who can achieve the complex goals of public service. Perry and Hondeghem (2008) believes that people prefer public service organizations because of job security, careers, pension systems, and development opportunities. Cao and Xi (2015) mainly focus on self-interest motivation, which attributes it to the higher status of civil servants in the social class, their occupational stability, and social security being superior to other social classes in China. In Russia, students rely on their parents’ advice more than their peers in other countries when making career choices. Parents working in the civil service are the most influential predictors of whether students would be interested in working in the public sector (Jkel and Borshchevskiy, 2018). Ciobanu and Androniceanu (2015) figure that generous salaries and a good working environment are important factors for people to choose public sector jobs. However, Druskienė and Šarkiūnaitė (2018) believe that the motivation system for civil servants should be balanced, considering the monetary, moral, political, and normative. They find that civil servants are more motivated by morality and norms than by politics and money. The inherent social value of the profession itself is also an important consideration when choosing to become a civil servant. Sattler and Kerr (1991) hold that high Social Value Orientation (SVO) people reveal a greater concern for others and the group. Moreover, they judge more in terms of non-egoistic values, such as fairness, honesty, and equality. The Public Service Motivation (PSM) theory proposed by scholars Perry and Wise (1990), which emphasizes social service, self-dedication, and realizing self-value in the social context. It provides a new perspective for the motivation of civil servants to apply for the examination. This theory is different from the “economic man” hypothesis, which focuses on maximizing their interests. It emphasizes the existence of individual altruistic behavior and public service spirit. It also believes that the higher the level of an individual’s public service motivation, the more likely he or she will seek to join a public organization. Motivation to perform public service can be seen as an emotional goal system that responds to social stimuli throughout life events and in institutional environments (Wang et al., 2020). In line with modern motivational theory in Psychology, SVO is seen as an individual trait (Grant, 2008). The attitudinal effect of high PSM is stronger if supported by a trait-driven “multiplier” of a pro-other SVO. That is, if an individual indicates she or he is motivated to serve the public interest (including a general motivation to self-sacrifice for the sake of others’ benefit), then this motivational attitude would be further boosted in case that the individual is characterized by the other-regarding trait of SVO as well (Waele et al., 2021). For this reason, this research is mainly based on the perspective of occupational values. Since the concept of occupational values was put forward in the last century, it has been widely concerned by scholars domestic and abroad and has formed relatively rich theoretical research results. Occupational values are considered to be important factors affecting occupational choices. Sparrow et al. (2010) point out that researchers from different disciplines use “work value,” “work ethic,” “work direction,” “work attitude,” and “work objectives” to represent work-related variables. Their basic meaning and connotation are generally consistent. With the deepening of the research, the connotation of occupational values has been continuously enriched, from the early Herzberg’s two-dimensional values (intrinsic value and extrinsic value), Ginzberg’s three-dimensional values (intrinsic value, extrinsic value, accompanying value), to the four-dimensional values (internal, external, social, and prestige) and even to the higher-dimensional values (Ben-Shem and Avi-Itzhak, 1991). By focusing on external values, researchers gradually introduce internal values, such as prestige value, social value, and other factors.

The current academic research on “civil servant fever” is mainly analyzed from economic, cultural, employment environment, and other factors. Firstly, research perspectives and conclusions mostly fall on government departments, universities and training units. Relevant research focuses on empirical inference and summary. Few scholars have studied whether college students who apply for civil servants have a high degree of public service motivation. Secondly, most studies lack clear theoretical frameworks and sufficient data support. Thirdly, the political system and cultural background of different countries also affect the dimension and understanding of public service motivation. Studies have shown that there are cultural differences not only in conceptual and operational definitions of public service motivation (Vandenabeele, 2008; Leisink and Steijn, 2009; Anderfuhren-Biget et al., 2010), but also in their causes and consequences. Kim and Kim (2016) found that the dimensions of different cultures do not have the same meaning and scale through the measurement and scale redevelopment of public service motivation in different countries. Even in countries with similar cultural values, languages, levels of development or geographic proximity, there may be identifiable patterns in public service motivations. Therefore, when conducting research, scholars of various countries need to modify the measurement tools to make them adapt to the unique cultural background of each country.

Public service motivation is considered an important factor influencing employment propensity in the public sector. This study incorporated it into the composition of occupational values. The research explored the correlation between college students’ “civil servant fever” and occupational values. Meanwhile, it introduced political efficacy as a mediating variable to clarify the relationship between occupational values, political efficacy, and college students’ willingness to apply for civil servants. This deepens the understanding of the motivation of college students’ willingness to apply for civil servants. To some extent, it would broaden the perspective of the study of “civil servant fever.” In addition, this research could help college students correctly examine their motivation to apply for civil servants. In addition, it encourages them to improve their public service motivation through their own efforts. Moreover, this study would provide theoretical basis and practical reference for college educators to implement college students’ career education.

## Literature review and theoretical hypothesis

### Occupational values and willingness to apply for civil servants

**Figure 1:**
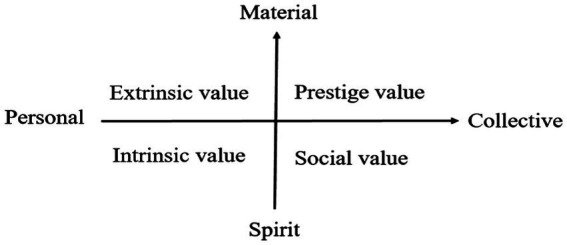
Classification of occupational values structure.

According to the theory of organizational behavior, values are the reflection of a series of basic beliefs of individuals (Stephen, 2011). Occupational values are the extension of individual values in the occupational dimension. They are the intrinsic needs of individuals to engage in occupation and the characteristics or attributes they pursue when engaging in occupational activities. Occupational values affect individual work motivation or attitude, and then affect individual performance, which is an effective way to motivate individual behavior (Elizur, 1996; Petroni and Colacino, 2008). Regarding the dimensional division and measurement of occupational values, Ronen (1993) formulates the criteria for the division of individual-collective and material-spiritual divisions. Accordingly, this study defines “occupational values” as an individual’s internal standard system for measuring the degree of matching among different organizations, job elements, and self-needs. This, in turn, influences the concept and position of individual career choices. In theory, referring to the division of the structure of occupational values by Ros et al. (1999), occupational values are divided into four dimensions, namely, intrinsic value, extrinsic value, social value, and prestige value from a macro perspective (Figure 1). Among them, regarding the relationship between public service motivation and occupational values, the study draws on the views of Choi (2017) and regards public service motivation as a part of social value in occupational values. Vandenabeele (2007) believe that public service motivation itself is a value that measures psychological tendencies. Therefore, public service motivation can be used to measure altruistic value in occupational values.

Previous studies have shown that occupational values play a key role in the process of career choice and are one of the factors that affect career decision-making self-efficacy (Lent et al., 1994; Zhou, 2015). Ravlin and Meglino (1987) point out that values are guidelines or standards for decision-making, and decisions reflect specific value patterns. Ben-Shem and Avi-Itzhak (1991) believe that occupational values are the main determinants of occupational choice. Judge and Bretz (1992) put forward that individuals are more inclined to choose jobs with value connotations similar to their value orientation. Based on the existing research, there might be a correlation between occupational values and employment intention, which, in turn, affects the willingness to apply for civil servants. Accordingly, this study proposes the following research hypotheses:

*H1*: Occupational values might affect the college students’ willingness to apply for civil servants.

### Occupational values and political efficacy

Occupational values play a key role in the process of career choice (Lent et al., 1994). The prestige value in occupational values is usually referred to as social status, prestige, etc. The resource preservation theory states that people always strive to obtain and maintain the resources they consider valuable. These resources include psychological capital, social support, achievement motivation, emotional stability, etc. (Hobfoll, 2001). Based on the person–organization (P-O) fit theory, scholars believe that people with higher public service motivation are more likely to choose careers in public sector organizations. They believe that their altruistic value orientation matches well with the working environment in the public sector (Kjeldsen, 2014). In addition, civil servants with high enthusiasm for public service can mobilize work resources more easily, optimize work demands and better serve the public (Bakker, 2015). Lee and Wilkins (2011) investigate the career motivation of employees in public sectors and non-profit institutions. As a result, they find that the career motivation of employees includes both external motivation factors (such as pursuing career development, salary, pension plan, and family-friendly policies), and internal motivation factors (such as work responsibility and desire to serve the public). According to Tepe and Vanhuysse (2017), public organizations are generally considered to provide a higher level of job security than private and non-profit organizations. Houston (2000) holds that intrinsic rewards are more valuable to public officials than extrinsic incentives (such as high income or short working hours). Jeannette (2005) holds that individuals engaged in public service may simply view their work as more about serving the community rather than gaining income and prestige. Moreover, the possibility to participate in the decision-making process increases job satisfaction, self-esteem, and identification with the organization of civil servants (Leisink and Steijn, 2008). The barriers to the public service are high in east Asian countries, such as China, South Korea, and Taiwan. The phenomenon is mainly rooted in the long-held belief that a career in civil service brings family privilege and prestige (Elman, 2013). In China, according to Cheng (2011), there is an important reason for the popularity of civil servants and civil servant positions. That is, the government has the power of social resources and social management. This gives civil servants specific control and management powers. Therefore, they have higher social status and power. Cao and Xi (2015) express similar views based on the perspective of social class and social mobility. They believe that the application of civil servants is very popular among college students. The main reason is the higher status of civil servants in the social class. It is superior to other social classes in terms of job stability and welfare security, and belongs to a relatively “decent” occupation. The expectation level of the graduates’ group’s prestige value reflects their willingness to participate in politics to a certain extent. That is, occupational values might affect the level of individual political efficacy. Accordingly, the following research hypotheses are proposed in this study:

*H2*: Occupational values might affect college students’ political efficacy.

### Political efficacy and willingness to apply for civil servants

Political efficacy refers to people’s perception that they can play a certain role in the decision-making process of the government. It is an individual’s psychological reflection of the relationship between the government, policies, and all political systems (Campbell et al., 1954). Political efficacy includes the public’s perception of the ability of their political actions to influence the political process and promote political and social change (Craig and Maggiotto, 1982). Gao and Huang (2018) think that political efficacy includes individuals’ cognition of their ability, as well as their judgment on whether the government and its public policies can be influenced. The higher the political efficacy of citizens, the easier it is to have a sense of trust in government (Kim, 2015). Scholars further divide political efficacy into two dimensions: internal political efficacy and external political efficacy. Internal political efficacy refers to citizens’ perception of their ability to understand politics or participate in political activities (Craig and Silver, 1990). External political efficacy refers to citizens’ views on the response of political institutions and actors to citizens’ needs (Converse, 1972; Balch, 1974). School is an important place for the socialization of civil politics. Through education, especially civic education, people learn the knowledge and skills needed to participate in politics and have a better understanding of their political environment (Hillygus, 2005). A large number of studies have shown that the level of education can have an important impact on political self-efficacy. Well-educated people might have a strong sense of political self-efficacy. The values and experience acquired by people through education are the main factors for the formation of political self-efficacy (Hayes and Bean, 1993; Verba et al., 1997; Warwick, 1998). Generally speaking, individuals with a strong sense of political efficacy are more likely to participate in political activities than those with a weak sense of political efficacy (Li, 2018). Gao and Huang (2018) find that political efficacy and public service motivation are effective factors to predict the motivation of college students to join the public sector. Moreover, political efficacy has a greater impact. Yan et al. (2017) believe that if citizens have confidence in their knowledge and ability to participate in politics, they will be more likely to participate in public affairs actively when the government can listen to their opinions. Accordingly, the following research hypotheses are proposed in this study:

*H3*: Political efficacy might affect the college students’ willingness to apply for civil servants.

### The mediating role of political efficacy

As mentioned above, occupational values have a certain influence on the college students’ willingness to apply for civil servants, and affect their self-efficacy in political participation. Numerous studies show that internal political efficacy plays a significant, positive role in political participation (Krampen, 2000; Condon and Holleque, 2013). Reichert (2016) examines the mediating role of internal political efficacy between political knowledge and political participation. The research also indicates that internal political efficacy increases intentions to participate politically. Therefore, political efficacy might be a factor affecting college students’ political participation. Occupational values can directly affect the willingness to apply for civil servants. It might also affect the political will and confidence of individuals to participate in political affairs by affecting their sense of political efficacy. Thereby, it makes individuals more actively involved in political affairs. This exerts its political influence in the public sector and field, which indirectly affects the willingness to apply for civil servants. Accordingly, this study believes that political efficacy might be a potential mediating variable between occupational values and college students’ willingness to apply for civil servants. Accordingly, the following hypotheses are proposed:

*H4*: Political efficacy might play a mediating role between occupational values and college students’ willingness to apply for civil servants.

**Figure 2:**
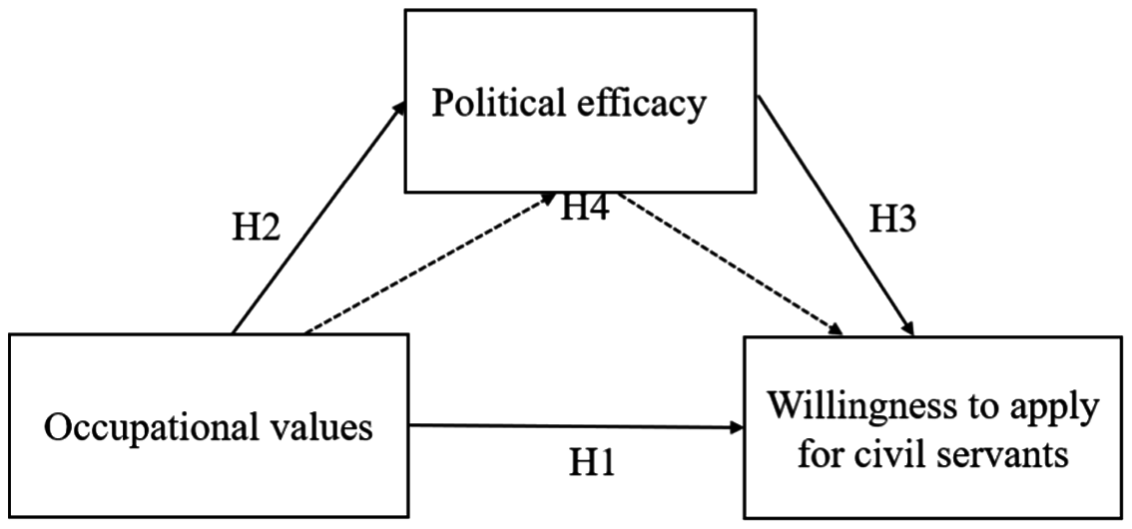
Research model diagram. H1: Occupational values → Willingness to apply for civil servants (solid line). H2: Occupational values → Political efficacy (solid line). H3: Political efficacy → Willingness to apply for civil servants (solid line). H4: Occupational values → Political efficacy → Willingness to apply for civil servants (dashed line).

To sum up, this study took college students studying in a “double first-class” university in China as the research object. It explored the relationship between college students’ occupational values, political efficacy, and willingness to apply for civil servants. As shown in Figure 2, the relationship model between them is proposed.

## Research method

On the basis of conducting an open-ended questionnaire survey and referring to relevant literature, this paper initially constructs a measurement system of college students’ willingness to apply for civil servants. The structural equation model technology and a certain amount of sample are used to conduct empirical research on the constructed index system. The constructed index system is revised and explained from a quantitative point of view. The results show that the established system has high stability, rationality, and wide applicability. Structural Equation Modeling (SEM) is a general linear statistical modeling technique. SEM presents the objective state of things in the form of causal assumptions, and then verifies it with quantitative data. It is an empirical analysis model method. By looking for the inherent structural relationship between variables, it can verify whether the assumption of a certain structural relationship or model is reasonable and whether the model is correct. In addition, it can also point out how to modify the problematic model. The sample size of this empirical study is relatively large. Thus, it can be seen from the exogenous variables that the sample distribution is reasonable and widely representative. Moreover, the model fitting results and re-checking validity are both good. It can be considered that the constructed index system has high rationality and wide applicability.

### Data source and sample characteristics

The convenience sampling method is adopted in this study. Since this method is extremely speedy, easy, readily available, and cost-effective, causing it to be an attractive option to most researchers (Henry, 1990). Five hundred and eighty questionnaires were distributed to the students of a double-first-class university in 2022. After sorting out, some samples with answer repetition rate above 70% were removed. Five hundred and sixty-six valid data were obtained, with an effective rate of 97.59%. Among them, 288 were undergraduates and 278 were postgraduates; 245 were male and 321 were female. The distributions of the samples in demographic variables such as gender, major, and household registration were relatively balanced, which could reduce the impact of the sample on the research results to a certain extent. Thus, the sample had a certain representativeness (Table 1).

**Table 1:** Basic characteristics of samples (*N* = 566).

Variable name		Sample size	Proportion
Gender	Male	245	43.3
	Female	321	56.7
Major	Social science	253	44.7
	Natural science	313	55.3
Educational level	Undergraduate	288	50.9
	Postgraduate	278	49.1
Household registration	Rural	276	48.8
	Town	121	21.4
	City	169	29.8

### Research measurement tools

The questionnaire had a total of 131 questions, and the KMO value was 0.956, showing good overall validity. The Cronbach’s α coefficient was 0.856, and the reliability met the requirements. Factors were extracted by factor analysis, and the factor loadings of all items were greater than 0.5, so all items were retained in the formal survey (Table 2).

**Table 2:** Theoretical construction of questionnaire dimension.

Primary dimension	Secondary dimension
Occupational values	Treatment development
	Career stability
	Intrinsic value
	Prestige value
	Working atmosphere
	Public service motivation
Political efficacy	Intrinsic efficacy
	External efficacy
Willingness to apply for civil servants	Employment tendency
	Exam preparation intensity

#### Occupational values scale

The topics of the occupational values scale mainly referred to the division of 15 items of occupational values (Super, 2007) and the research on the structure of occupational values of college students and the new generation (Jin and Li, 2005; Hou et al., 2014). There were 29 questions on this scale. Exploratory factor analysis of the scale showed that the KMO value was 0.957, indicating that the questionnaire data were suitable for extracting information. In the exploratory factor analysis, there were six factors with an eigenvalue greater than 1, which jointly explained 81.717% of the total variation of the occupational value scale. They were named as treatment development, career stability, intrinsic value, prestige value, working atmosphere and public service motivation, respectively. Comparing the structural division in the research design, treatment development and career stability belonged to the extrinsic value dimension; working atmosphere and public service motivation belonged to the social value dimension; and the division of intrinsic value and prestige value was the same as the original hypothesis. After removing some items with low factor load coefficients, the treatment development included four items such as “getting a higher salary” and “good industry prospects,” etc. Career stability included four items such as “low work pressure” and “good job stability,” etc. Intrinsic value included four items such as “work and personality,” “work is creative and diverse,” etc. Prestige value included three items such as “being able to gain social approval and social recognition,” “has a high social status,” etc. The working atmosphere included four items such as “being able to work with friendly and mutually helpful colleagues” and “organic culture with clean air,” etc. Public service motivation included eight items such as “meaningful public welfare activities are important to me,” “When I see other people encountering difficulties, I feel bad,” etc. The design was carried out on a 5-point Likert scale, with “1” indicating “complete disagreement” and “5” indicating “complete agreement.” In addition, public service motivation was one of the sub-dimensions of the occupational value scale. It was mainly derived from Perry’s four-dimensional model of Attraction to Policy Making (APM), Commitment to the Public Interest (CPI), Compassion (COM), Self-Sacrifice (SS), and adjusted in combination with the actual domestic situation (Perry, 1996). The Cronbach’s α coefficient of the occupational values scale was 0.949. In addition, the Cronbach’s α coefficient of each sub-dimension was greater than 0.8.

#### Political efficacy scale

Political efficacy included internal efficacy and external efficacy. Political efficacy was measured by using the 2010 Chinese General Social Survey (CGSS), which was an internationally accepted measure of political efficacy (Zhang et al., 2017). The scale included five items such as “I feel I have the ability to participate in politics,” “When I make suggestions to government agencies, they will be adopted by the relevant departments,” etc. The design was carried out on a 5-point Likert scale, with “1” indicating “strongly disagree” and “5” indicating “strongly agree.” The Cronbach’s α coefficient of the political efficacy scale was 0.938.

#### Willingness to apply for civil servants scale

The willingness to apply for the civil servants scale included the individual’s willingness to choose the civil servant industry as an employment position and the willingness to make efforts for it (Blau, 1994). Considering college students’ tendency to apply for civil servants, the scale included two sub-dimensions, employment tendency, and exam preparation intensity. The scale included 10 items such as “I will take the initiative to apply for civil servants,” “I will spend a long time preparing for civil servants,” etc. The design was carried out on a 5-point Likert scale, with “1″ indicating “strongly disagree” and “5″ indicating “strongly agree.” The Cronbach’s α coefficient of willingness to apply for civil servants scale was 0.948.

### Data processing

To test the reliability of each scale and the discriminant validity of each variable, SPSS 26.0 was used for descriptive statistical analysis, difference test, correlation analysis, and common method deviation. Amos24.0 plug-in was used to verify the hypothesis model. Also, the structural equation model and bias-corrected bootstrap were used to verify the hypothesis.

## Research results

### Confirmatory factor analysis

AMOS24.0 was used for confirmatory factor analysis (CFA) to test the validity of all variables, including the occupational values (six dimensions), political efficacy and willingness to apply for civil servants. The results are shown in Table 3. In each measurement relationship, the absolute value of standardized load system was greater than 0.6 and showed significance, indicating that the scale had a good measurement relationship. The combined reliability (CR) of each variable ranged from 0.772 to 0.926, all greater than 0.70. The average variation (AVE) was also close to or greater than 0.6. The results showed that the above variables had good convergence validity (Schermelleh-Engel et al., 2003).

**Table 3:** Results of reliability and validity tests.

Variables	Items	Std. error	*p*	Std. estimate	AVE	CR
Intrinsic value	Q10	–	–	0.832	0.649	0.902
	Q11	0.043	0.000	0.885		
	Q12	0.046	0.000	0.842		
	Q13	0.051	0.000	0.706		
	Q14	0.047	0.000	0.751		
Public service	APS	–	–	0.877	0.758	0.926
	CPV	0.029	0.000	0.843		
	COM	0.029	0.000	0.888		
	SS	0.030	0.000	0.874		
Career stability	Q3	–	–	0.812	0.710	0.907
	Q4	0.039	0.000	0.826		
	Q5	0.040	0.000	0.865		
	Q6	0.039	0.000	0.866		
Working atmosphere	Q15	–	–	0.863	0.782	0.935
	Q16	0.035	0.000	0.885		
	Q17	0.035	0.000	0.908		
	Q18	0.037	0.000	0.879		
Prestige value	Q19	–	–	0.885	0.539	0.772
	Q20	0.043	0.000	0.719		
	Q21	0.050	0.000	0.603		
Treatment development	Q1	–	–	0.854	0.740	0.919
	Q2	0.033	0.000	0.871		
	Q7	0.032	0.000	0.859		
	Q8	0.036	0.000	0.857		
Political efficacy	Intrinsic efficacy	–	–	0.869	0.776	0.874
	External efficacy	0.050	0.000	0.892		
Willingness to apply	Employment tendency	–	–	0.655	0.663	0.792
	Exam preparation intensity	0.110	0.000	0.947		

HTMT (heterotrait-monotrait ratio) was used to test the discriminant validity. The results are shown in Table 4. The value in the table represented the HTMT value between two factors. Generally, HTMT value less than 0.85 indicated discriminant validity between the two factors. All the HTMT values in the table were within the standard range, indicating that the data had good discriminative validity.

**Table 4:** HTMT (heterotrait-monotrait ratio) test.

	Intrinsic value	Public service	Career stability	Working atmosphere	Prestige value	Treatment development	Political efficacy	Willingness to apply
Intrinsic value	–							
Public service	0.766	–						
Career stability	0.590	0.603	–					
Working atmosphere	0.829	0.789	0.668	–				
Prestige value	0.807	0.804	0.628	0.718	–			
Treatment development	0.752	0.719	0.829	0.801	0.720	–		
Political efficacy	0.363	0.470	0.277	0.237	0.470	0.316	–	
Willingness to apply	0.304	0.551	0.419	0.313	0.528	0.286	0.644	–

### Correlation analysis and regression analysis

#### Overall analysis

##### Correlation analysis

The Pearson’s coefficient correlation analysis between the variables is shown in Table 5. The six dimensions of occupational values and political efficacy were significantly positively correlated with the college students’ willingness to apply for civil servants. The results were in line with theoretical expectations and need to be verified by regression analysis and the construction of structural equation models.

**Table 5:** Correlation analysis of Pearson’s coefficient of each variable.

	Willingness to apply	Treatment development	Career stability	Intrinsic value	Prestige value	Working atmosphere	Public service	Political efficacy
Willingness to apply	1							
Treatment development	0.240^**^	1						
Career stability	0.346^**^	0.740^**^	1					
Intrinsic value	0.253^**^	0.679^**^	0.522^**^	1				
Prestige value	0.416^**^	0.611^**^	0.524^**^	0.682^**^	1			
Working atmosphere	0.266^**^	0.739^**^	0.606^**^	0.759^**^	0.614^**^	1		
Public service	0.467^**^	0.658^**^	0.542^**^	0.698^**^	0.688^**^	0.733^**^	1	
Political efficacy	0.547^**^	0.290^**^	0.250^**^	0.336^**^	0.408^**^	0.221^**^	0.440^**^	1

* means *p* < 0.05:

** means *p* < 0.01:

*** means *p* < 0.001.

##### Multiple linear regression analysis

The control variables were filtered. Demographic variables included gender, educational level, major, and household registration. The difference test on the dependent variable (including independent sample *T*-test and one-way ANOVA test) was carried out for these four variables. The analysis results are shown in Table 6. It was shown that there was no significant difference between genders in the willingness to apply for civil servants. However, there were significant differences in the willingness to apply for other variables. Therefore, educational level, major, and household registration were selected as control variables to remove the influence of interference factors on the analysis results.

**Table 6:** Difference test.

Variable	Classification	Willingness to apply	T/F	*P*
Gender	Male	3.214 ± 0.792	1.1019	0.309
	Female	3.144 ± 0.816		
Educational level	Postgraduate	3.321 ± 0.827	−0.3984	0.000
	Undergraduate	3.054 ± 0.757		
Major	Social Science	3.299 ± 0.814	3.134	0.000
	Natural science	3.086 ± 0.780		
Household registration	Rural	2.823 ± 0.778	3.690	0.026
	Town	3.058 ± 0.947		
	City	2.991 ± 0.957		

Multiple linear analysis refers to the use of multiple independent variables to predict or explain a dependent variable. In this research, a regression equation was used to verify the influence of motivation on the willingness to apply for civil servants. In this study, the six dimensions of professional values were used as independent variables in the regression analysis. After dealing with dummy variables, educational level, major, and household registration were used as control variables. In addition, this study used the willingness to apply for civil servants as the dependent variable. Moreover, the multiple linear regression method was used to test, so as to explore the factors affecting the willingness to apply for civil servants. The multiple regression results are shown in Table 7.

**Table 7:** Linear regression analysis results.

Model			Non-standardized coefficient		Standard coefficient	*t*	*P*	*VIF*
			*Beta*	Standard error	*Beta*			
Constant			1.080	0.187		5.779	0.000	
Independent variable	Treatment development		−0.301	0.072	−0.269	−4.155	0.000	3.433
	Career stability		0.310	0.057	0.289	5.446	0.000	2.299
	Intrinsic value		−0.148	0.066	−0.138	−2.259	0.024	3.033
	Working atmosphere		−0.152	0.068	−0.145	−2.228	0.026	3.443
	Prestige value		0.270	0.058	0.251	4.670	0.000	2.366
	Public service motivation		0.570	0.066	0.508	8.637	0.000	2.819
Control variable	Educational level	Postgraduate	0.202	0.062	0.126	3.262	0.001	1.216
		Undergraduate	0					
	Major	Social science	0.045	0.062	0.028	0.717	0.473	1.226
		Natural science	0					
	Household registration	Rural	−0.122	0.073	−0.076	−1.680	0.094	1.678
		Town	−0.048	0.080	−0.028	−0.602	0.547	1.717
		City	0					
*R^2^*					0.332			
*Adjusted R^2^*					0.320			
*F*					27.146			
*P*					0.000			

Dependent variable: Willingness to apply for civil servants.

After multiple regression analysis, it was shown that the six variables of occupational values had a significant impact on the willingness to apply for civil servants by excluding the confounding interference of educational level, major, and household registration. The R-squared value was 0.330, which showed that these dimensions explained 33.0% of the change in the willingness to apply for civil servants. The model passed the F test (*F* = 27.146, *p* = 0.000 < 0.05), indicating that the model was valid. In addition, through the multicollinearity test of the model, it was found that the VIF values in the model were less than 5. This meant that there was no collinearity problem and there was no correlation between the sample data. The model was good.

**Figure 3:**
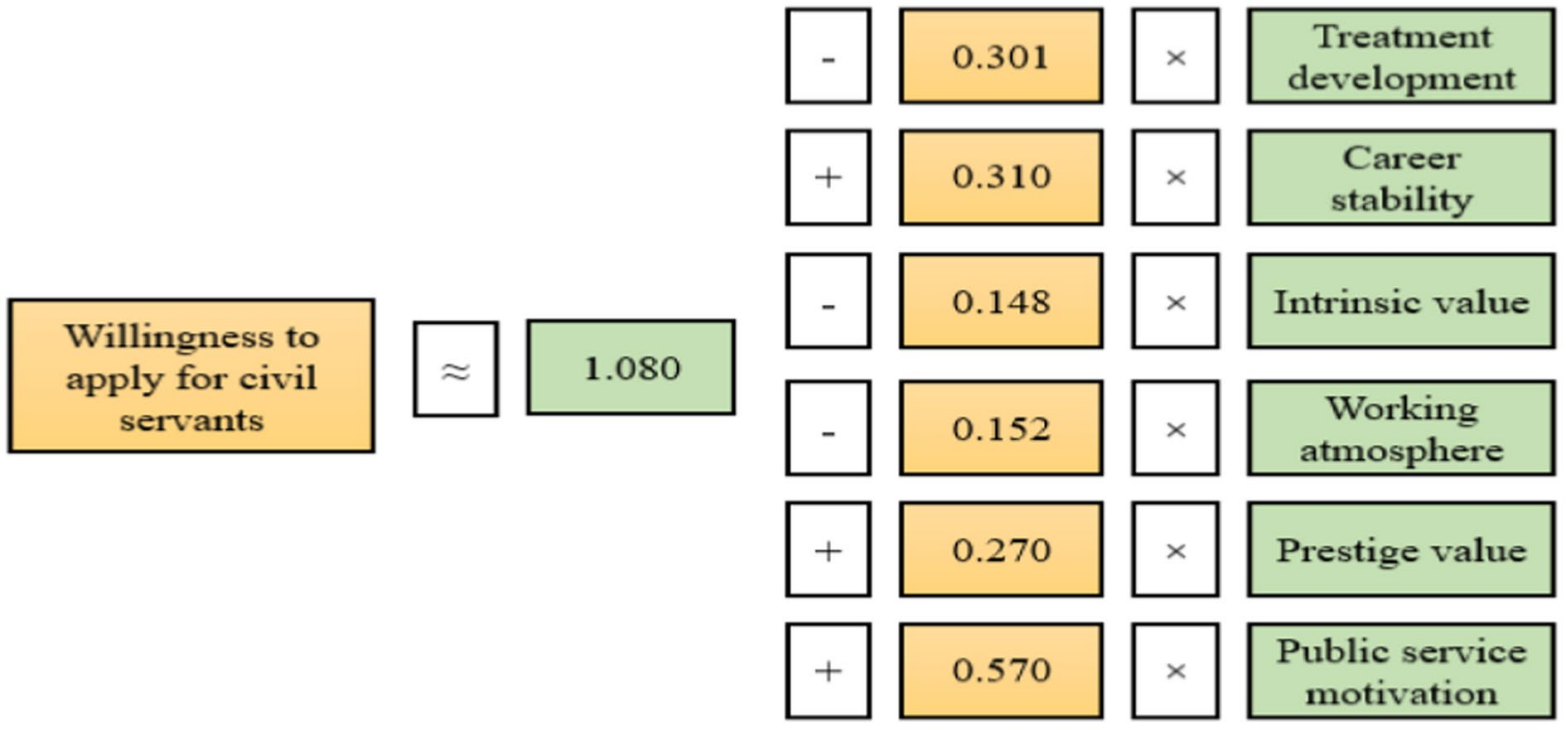
Diagram of stepwise regression model.

**Model formula:** Willingness to apply for civil servants = 1.080–0.301 × Treatment development + 0.310 × Career stability − 0.148 × Intrinsic value − 0.152 × Working atmosphere + 0.270 × Prestige value + 0.570 × Public service motivation. The stepwise regression model was shown in Figure 3.

#### Dimensional analysis

##### Correlation analysis

In the overall analysis, the public service motivation had a significant positive impact on the willingness to apply for civil servants. Therefore, this study further selected the dimension of public service motivation to explore its influence on the willingness of college students to apply for civil servants. The Cronbach’s α coefficient of the Public Service Motivation Scale was 0.970, among which the Cronbach’s four sub-dimensions of APM, CPI, COM, and SS was higher than 0.8 (Table 8). The Pearson’s correlation coefficient diagram of the sub-dimensions of the willingness to apply for civil servants and public service motivation is shown in Table 9. It indicated that the dimension scale had good consistency in measuring these four dimensions, and each dimension was significantly related to the willingness to apply for civil servants.

**Table 8:** Sub-dimensional Cronbach’s α coefficients.

	APM	CPI	COM	SS
Cronbach’s α	0.848	0.938	0.944	0.941

**Table 9:** Pearson’s correlation coefficient.

	Willingness to apply	APM	CPI	COM	SS
Willingness to apply	1				
APM	0.303^**^	1			
CPI	0.284^**^	0.709^**^	1		
COM	0.296^**^	0.726^**^	0.780^**^	1	
SS	0.338^**^	0.805^**^	0.738^**^	0.817^**^	1

* means *p* < 0.05;

** means *p* < 0.01;

*** means *p* < 0.001.

##### Multiple linear regression analysis

**Figure 4:**
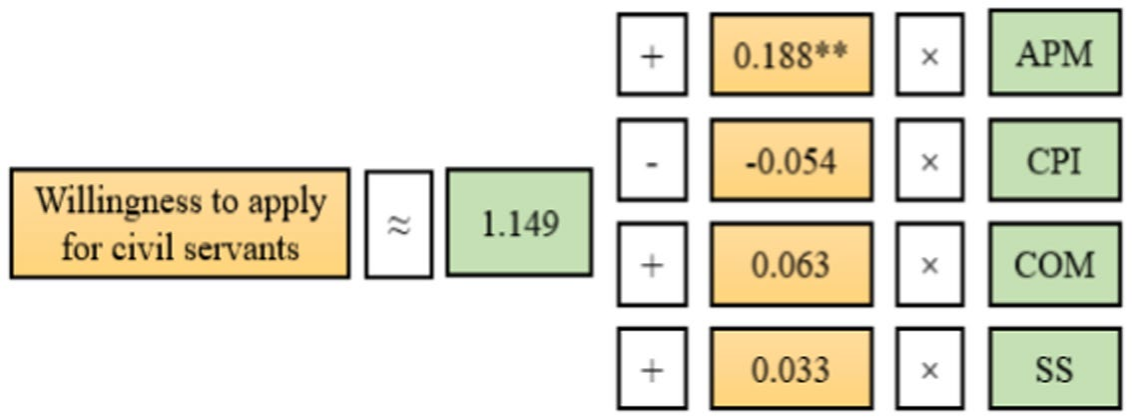
Multiple linear regression model.

A linear regression analysis model was established for the willingness to apply for civil servants. The APM, CPI, COM, and SS were selected as variables, and they were calculated by the Likert Scale to establish the regression analysis model. The regression model results are shown in Table 10 and Figure 4.

**Table 10:** Results of linear regression analysis.

Variable	Non-standardized coefficient		Standard coefficient	*t*	*P*	*VIF*	*R^2^*	Adjusted *R^2^*	*F*
	*Beta*	Standard error	*Beta*						
Constant	1.149	0.281	−	4.095	0.000^**^	−	0.047	0.040	*F*(4,551) = 6.724 *p* = 0.000
APM	0.188	0.062	0.200	3.036	0.003^**^	2.509			
CPI	−0.054	0.090	−0.054	−0.063	0.547	4.669			
COM	0.063	0.081	0.055	0.783	0.434	2.809			
SS	0.033	0.088	0.031	0.375	0.708	4.049			

The APM, CPI, COM, and SS were taken as independent variables. The willingness to apply for civil servants was taken as a dependent variable for linear regression analysis. The R^2^ value of the model was 0.047, and these independent variables explained 4.7% of the change in willingness to apply for civil servants. F-test was conducted on the model. It was found that the model passed the F-test (*F* = 6.724, *p* = 0.000 < 0.05), indicating that at least one of the four variables would have an impact on the willingness to apply for civil servants. In addition, the multicollinearity test of the model showed that VIF values in the model were all less than 5. This indicated that there was no collinearity problem in the model. Moreover, the value of D-W was near the number 2. This indicated that there was no autocorrelation in the model, and there was no correlation between the sample data. Thus, the model was good. The results showed that APM had a significant positive impact on the willingness to apply for civil servants. However, the CPI, COM, and SS did not have an impact on the willingness to apply for civil servants.

**Model formula:** Willingness to apply for civil servants = 1.149 + 0.188 × APM-0.054 × CPI +0.063 × COM +0.033 × SS.

The multiple linear regression model was shown in Figure 4.

### Hypothesis testing

The structural equation model was used to further test the influence of occupational values on the willingness to apply for civil servants. Taking the six dimensions of occupational values as independent variables, the willingness to apply for civil servants as the dependent variable, and the political efficacy as the mediating variable, the model was established and tested. Occupational values were divided into 6 dimensions: treatment development, career stability, intrinsic value, prestige value, working atmosphere, and public service motivation. Among them, treatment development and career stability belonged to the extrinsic value dimension; working atmosphere and public service motivation belonged to the social value dimension. Political efficacy was divided into two dimensions: internal efficacy and external efficacy. In addition, willingness to apply for civil servants included two dimensions of employment tendency and exam preparation intensity. Four structural equation models (intrinsic value, prestige value, extrinsic value, and social value) were constructed, respectively. The data analysis results showed that the factor loadings of each index were significant (*p* < 0.001), indicating that each latent variable was well represented by its index.

**Figure 5:**
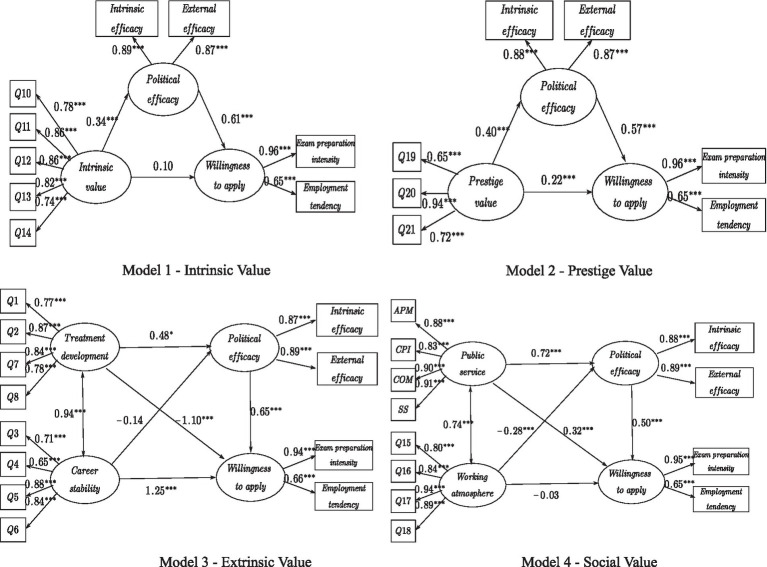
Four models of structural equations. * means *p* < 0.05, ** means *p* < 0.01, *** means *p* < 0.001.

The equation model was calculated according to the mediation effect test procedure proposed by Wen et al. (2004). The results were shown in Figure 5, and the model fitting results are shown in Table 11. The model fitting results were all good, indicating that the hypothetical model and the collected data could be well matched.

**Table 11:** Fitting indicators of each mediation model.

Model	χ^2^/df	RMSEA	GFI	NFI	RFI	IFI	TLI
Model 1	2.561	0.053	0.980	0.983	0.969	0.989	0.981
Model 2	3.499	0.067	0.983	0.980	0.959	0.986	0.970
Model 3	2.561	0.053	0.980	0.983	0.969	0.989	0.981
Model 4	3.304	0.064	0.957	0.975	0.962	0.983	0.973

From the structural equation model, it was concluded that the prestige value of occupational values, career stability, public service motivation, and treatment development dimensions had significant impact on the willingness to apply for civil servants. This further supported Hypothesis H1. That was, occupational values significantly affected the willingness to apply for civil servants. For hypothesis H2, the path coefficients of the influence of political efficacy on the willingness to apply for civil servants were all significant. This supported Hypothesis H2. That was, political efficacy significantly affected the willingness to apply for civil servants. According to hypothesis H3, except for the dimension of occupational stability, the other five dimensions of occupational values had a significant influence on path regression coefficients on political efficacy. This supported Hypothesis H3.That was, occupational values would affect political efficacy.

The widely accepted bias-corrected bootstrap method was used to test the mediating role of political efficacy between occupational values and willingness to apply for civil servants. AMOS24.0 was used to set the bootstrap to 5000 times, and the confidence interval was 95% for the mediating effect test. The results are shown in Table 12. In path 1 and path 6, the confidence interval of indirect effect did not contain 0, while the confidence interval of direct effect contained 0. These indicated that political efficacy had a complete mediating effect on intrinsic value, working atmosphere and willingness to apply for civil servants. In path 2 and path 5, the confidence intervals of indirect effect and direct effect did not contain 0. This indicated that political efficacy had a partial mediating effect on prestige value, public service motivation and willingness to apply for civil servants. In path 3 and path 4, the confidence interval of indirect effect contained 0. This indicated that there was no mediating effect of political efficacy between extrinsic value and willingness to apply for civil servants. Thus, based on the above analysis, hypothesis H4 was verified. That was, political efficacy played a mediating role between occupational values and willingness to apply for civil servants.

**Table 12:** Mediation effect test.

Mediation model	Path	Effect type	Estimate	95% Confidence interval		Mediation type
				Lower limit	Upper limit	
Intrinsic value	Path 1—Intrinsic value	Indirect effect	0.281	0.266	0.561	Fully mediation
		Direct effect	0.131	−0.012	0.290	
		Total effect	0.417	0.727	0.940	
Prestige value	Path 2—Prestige value	Indirect effect	0.293	0.195	0.413	Partial intermediary
		Direct effect	0.287	0.131	0.442	
		Total effect	0.582	0.434	0.718	
Extrinsic value	Path 3—Career stability	Indirect effect	−0.108	−1.083	0.477	None
		Direct effect	1.493	0.805	3.788	
		Total effect	1.386	0.690	3.237	
	Path4—Treatment development	Indirect effect	0.419	−0.022	1.598	None
		Direct effect	−1.456	−4.032	−0.671	
		Total effect	−1.037	−2.590	−0.253	
Social value	Path 5—Public service motivation	Indirect effect	0.428	0.308	0.581	Partial intermediary
		Direct effect	0.376	0.158	0.588	
		Total effect	0.804	0.602	1.000	
	Path6—Working atmosphere	Indirect effect	−0.159	−0.249	−0.081	Fully mediation
		Direct effect	−0.029	−0.198	0.141	
		Total effect	−0.188	−0.371	−0.007	

## Conclusion

### Brief conclusion

This research explored the relationship between occupational values and college students’ willingness to apply for civil servants while verifying the mediating role of political efficacy. The results confirm that: (1) Occupational values positively affect the college students’ willingness to apply for civil servants. (2) Occupational values positively affect college students’ political efficacy. (3) Political efficacy positively affects the college students’ willingness to apply for civil servants. (4) Political efficacy plays a partial mediating role in the transmission path of occupational values and willingness to apply for civil servants.

### Discussion

#### The relationship between occupational values, political efficacy, and willingness to apply for civil servants

##### Occupational values affect the college students’ willingness to apply for civil servants

The above research showed that public service motivation had positive impact on the willingness to apply for civil servants. It indicated that college students attached great importance to the positive impact of a civil service career on factors such as reflecting social value, realizing life dreams, realizing political pursuit, and making contributions to society. Homberg et al. (2015) believe that individuals with high public service motivation will show higher job satisfaction when they have more opportunities to serve the public. Those inclined to public engagement were particularly drawn to become civil servants (Corey and Garand, 2002; Brewer, 2003). The second was career stability, which indicated that compared with other occupations, civil servants were more favored by college students. Bellante and Link (1981) confirmed that people who are more risk averse are more likely to work in the public sector. They value the stability of their employment. Irsutami and Sitepu (2018) find that stabilized future and a secure environment are important factors for undergraduates to become civil officers according to the research of 268 university students. The last was the prestige value, which showed that the social status, administrative power, and social recognition of the civil servant career were also important influencing factors for college students to apply for civil servants. It was worth noting that the dimension of salary development had a significant negative impact on the college students’ willingness to apply for civil servants. Individuals with high requirements for salary development were relatively less willing to apply for civil servants. This show that under the national policy of anti-corruption and a good social atmosphere in China, the income and benefits of civil servants tend to be open and transparent. Compared with other industries, especially the talent-intensive emerging industries dominated by high-tech manufacturing and internet companies, the civil service compensation system has no significant advantages. College students who pursue higher salaries and career development prospects have relatively low willingness to apply for civil servants.

In addition, intrinsic value and working atmosphere also negatively affected the college students’ willingness to apply for civil servants to a certain extent. Individuals with higher requirements for job creativity, challenge, and interest matching were less likely to apply for the civil servants. It showed that some college students still had the stereotype of “rigid” and “bureaucratic” on the nature of civil servants’ work. It was difficult to attract college students who pursue a relaxed and harmonious working atmosphere. Payment and working conditions are important predictors of public sector employees’ motivation, work effectiveness, and involvement (Serhan et al., 2018). Therefore, the salary should be increased and a good working environment should be created to make the job more attractive. Since the dimension of public service motivation had the most significant influence on occupational values, as a breakthrough point, it was believed that among the four sub-dimensions of public service motivation, political participation willingness was an important factor affecting college students to apply for civil servants. Under the background of the increasingly perfect domestic social and political democratic governance, how to correctly guide college students to participate in politics and improve their level of political participation is an urgent problem to be solved. In addition, although the three dimensions of public interest identification, compassion, and self-sacrifice had no significant impact on college students applying for civil servants, the positive spiritual value contained in them still played an important role in shaping college students’ complete personalities, consolidating their firm political stance and improving their future on-the-job governance ability.

##### Occupational values affect college students’ political efficacy

The results of the structural equation model analysis showed that in addition to occupational stability, the other five dimensions of occupational values significantly affected political efficacy. Among them, the working atmosphere negatively predicted political efficacy, indicating that individuals who valued the working atmosphere more were pursuing a relaxed and harmonious work environment, rather than pursuing personal political value realization. Public service motivation, treatment development, intrinsic value, and prestige value positively predicted political efficacy. It reflected that individuals who were full of public spirit and who pursued development space, self-realization, and value social status had a higher sense of political efficacy. Studies have shown that public service motivation can improve the work attitude and performance of public sector workers. Moreover, public service motivation is significantly related to career well-being and job satisfaction (Zheng and Zhou, 2017). According to the influence of occupational values and political efficacy, colleges could adjust career guidance strategies according to personal conditions and employment environment, and carry out career planning correctly. Moreover, colleges should guide students to carry out self-ability exploration correctly, and guide them to look at the employment situation objectively. Avoid the blind pursuit of self-efficacy, which leads to the phenomenon of “high vision but low hand,” “unfit for a higher post but unwilling to take a lower one.”

##### Political efficacy affects the willingness to apply for civil servants

Political efficacy includes an individual’s cognition of one’s abilities, as well as an individual’s judgment on whether the government and its public policies can be influenced (Gao and Huang, 2018). The above research showed that the individual’s sense of political efficacy had a significant positive impact on the willingness to apply for civil servants. Individuals with a stronger sense of political efficacy were more aware of their ability to participate in and influence the formulation of public policies. In addition, they were more willing to participate in the governance of governments at all levels, which reflected the value concept of “achieving success while benefiting the world” to a certain extent. Chetkovich (2003) finds that students with government employment intentions are more motivated by “opportunities to make social contributions” than those with private sector employment intentions. Compared to individuals who work in the private sector, civil servants are more likely to vote, donate, and participate in social and political activities (Brewer, 2003; Ertas, 2015). Regardless of whether they intend to join the government, most of the college students express their desire to “make some changes” in the questionnaire item on political efficacy. Zhang et al. (2016) found that political efficacy could positively promote citizens’ political participation behavior. In addition, it enables individuals to strengthen their beliefs in the process of political participation, and builds a virtuous circle between citizens’ external political participation behavior and internal political efficacy. To sum up, apart from stable careers and good welfare, providing space for college students to display their ambitions is also an important dimension that government agencies attract outstanding college students. This requires the governments to improve its own governance level. Governments should improve the selection mechanism within the system and its governing environment, so as to attract more outstanding college students to join.

#### The mediating role of political efficacy

In the analysis of the impact path of occupational values, including public service motivation and college students’ willingness to apply for civil servants, a structural equation model study was conducted by using political efficacy as a mediating variable. The results showed that political efficacy played a mediating variable in the relationship between the intrinsic value, prestige value, social value of occupational values and the willingness to apply for civil servants. However, there was no mediating effect on the path of extrinsic value-political self-efficacy and willingness to apply for civil servants. Occupational values not only directly affected the willingness to apply for civil servants, but also indirectly affected the willingness to apply for civil servants by influencing political efficacy. In the study, there was no mediating effect between the extrinsic value of college students’ political efficacy and the willingness to apply for civil servants. The reason might be that extrinsic value mainly focused on future career development, stability and treatment. However, the measurement dimension of the political efficacy scale focused on personal political will and perception of their political efficacy, which had no theoretical causal relationship with extrinsic value dimension. While the measurement of the extrinsic value dimension represented the most real and most typical personal value pursuit of the group. The groups that attached more attention to the extrinsic value dimension pursued high salaries and paid attention to rapid promotion and growth, which was also an important part of personal values and needed to be further guided correctly.

### Contribution

#### Theoretical contributions

The theoretical contributions of this study are as follows: Firstly, the research on the relationship between occupational values and political efficacy is enriched. At present, there are few related types of research in China. This study explores the influence of occupational values on college students’ willingness to apply for civil servants. And it enriches the related research on occupational values in the field of career development, and further promotes the localization of occupational values-political efficacy. Secondly, the relationship between occupational values and college students’ “civil servant fever” phenomenon is further analyzed. This study uses political efficacy to explain the mechanism of occupational values on college students’ willingness to apply for civil servants. Then, the research emphasizes the mediating effect of political efficacy, and clarifies the logical relationship between occupational values and civil servants’ willingness, which further enriches the research in the field of college students’ career development. Third, due to the dependence of occupational values on the cultural and social environment, scholars in different countries need to modify the measurement tools when researching them, to adapt them to the unique cultural background of each country. The questionnaire used in this study was adjusted by Chinese scholars in combination with China’s actual cultural background based on referring to the scale development experience of internationally renowned scholars. This research will, to a certain extent, arouse further attention of domestic and foreign scholars on the phenomenon of college students applying for civil servants. Thereby, this research will enrich a deeper theoretical database in the field of career development. In addition, the research results have a certain analytical effect on revealing the fever of domestic and foreign college students applying for civil servants during the normalization of the epidemic. In the career development education of college students, the role of political efficacy between occupational values and the willingness to apply for civil servants should not be ignored.

#### Implication

With the increasing pressure of economic development, the continuous increase in the scale of graduates, and the continuous impact of the epidemic on employment, the employment concept of college students is constantly changing. College students, as an employer group with high comprehensive quality, are high-quality human resources to enrich the national civil servants. At present, the enthusiasm of college students to apply for civil servants is the result of joint efforts. On the one hand, the job is stable, which has certain social status, security in training, medical care, retirement, and so on. The COVID-19 pandemic has strengthened college students’ recognition of it. On the other hand, under the influence of the traditional concept of “excellence in learning leads to an official career” in China, some college students regard civil servants as a yardstick for measuring social status and a standard for realizing the value of life. The polarization phenomenon in the current civil servant application also reflects the current part of college students’ utilitarian and blind choice of career. Some college students apply for civil servants with strong utilitarian thought. Repeatedly applying for the exam not only causes a certain waste of social resources, but also wastes the precious youth of college students who have not been admitted to the civil servants for many years. Therefore, under the background of the civil servant examination fever, all sectors of society must return to rational thinking.

### Limitation

This study still has limitations. Firstly, due to the limited cross-sectional data sources, the sample has certain limitations, which are still insufficient in the confirmation of variable causality inference. Secondly, in the case of convenience sampling, there might be selection bias and potential threats. Finally, due to time constraints, the questionnaire was conducted without intervention due to time constraints. Therefore, as part of future research, follow-up studies should be designed and conducted using multiple data collection methods. Longitudinal studies should be conducted after conducting questionnaires. Future studies could use longitudinal data to verify the causal relationship of correlated variables.

## Data availability statement

The original contributions presented in the study are included in the article/supplementary material, further inquiries can be directed to the corresponding author.

## Author contributions

JN and CC designed the study and wrote the manuscript. YS and XL analyzed the data. CC and JN modified the manuscript. CC supervised the development of research and provided funding support. All authors contributed to the article and approved the submitted version.

## Funding

This study received funding from the Youth Project on Education supported by the National Social Science Fund of China “Research on the motivation and guarantee mechanism of enterprises’ participation in school running under the mixed ownership reform of higher vocational colleges” (CIA220278).

## Conflict of interest

The authors declare that the research was conducted in the absence of any commercial or financial relationships that could be construed as a potential conflict of interest.

## Publisher’s note

All claims expressed in this article are solely those of the authors and do not necessarily represent those of their affiliated organizations, or those of the publisher, the editors and the reviewers. Any product that may be evaluated in this article, or claim that may be made by its manufacturer, is not guaranteed or endorsed by the publisher.
